# Amplicon-based metagenomics identified candidate organisms in soils that caused yield decline in strawberry

**DOI:** 10.1038/hortres.2015.22

**Published:** 2015-06-03

**Authors:** Xiangming Xu, Thomas Passey, Feng Wei, Robert Saville, Richard J. Harrison

**Affiliations:** 1 East Malling Research, East Malling, West Malling, Kent, ME19 6BJ, UK

## Abstract

A phenomenon of yield decline due to weak plant growth in strawberry was recently observed in non-chemo-fumigated soils, which was not associated with the soil fungal pathogen *Verticillium dahliae*, the main target of fumigation. Amplicon-based metagenomics was used to profile soil microbiota in order to identify microbial organisms that may have caused the yield decline. A total of 36 soil samples were obtained in 2013 and 2014 from four sites for metagenomic studies; two of the four sites had a yield-decline problem, the other two did not. More than 2000 fungal or bacterial operational taxonomy units (OTUs) were found in these samples. Relative abundance of individual OTUs was statistically compared for differences between samples from sites with or without yield decline. A total of 721 individual comparisons were statistically significant – involving 366 unique bacterial and 44 unique fungal OTUs. Based on further selection criteria, we focused on 34 bacterial and 17 fungal OTUs and found that yield decline resulted probably from one or more of the following four factors: (1) low abundance of *Bacillus* and *Pseudomonas* populations, which are well known for their ability of supressing pathogen development and/or promoting plant growth; (2) lack of the nematophagous fungus (*Paecilomyces* species); (3) a high level of two non-specific fungal root rot pathogens; and (4) wet soil conditions. This study demonstrated the usefulness of an amplicon-based metagenomics approach to profile soil microbiota and to detect differential abundance in microbes.

## Introduction

*Verticillium dahliae* Kleb. is a soil-borne fungal pathogen, which penetrates the roots of a wide range of host plant species causing the disease Verticillium wilt^1^. The pathogen colonises the vascular system of the roots and crown depriving the leaves and stems of water. Also, it produces microsclerotia in the host as the infected tissues senesce, which are released into the soil as the plant decays and are the primary inoculum of *V*. *dahliae* in the soil for subsequent infection. Microsclerotia may survive for more than 10 years in the soil in the absence of its hosts^1^. Wilt incidence tends to be higher in soils infested with the root lesion nematode, *Pratylenchus penetrans*, which feed on the roots causing wounds, thus increasing entry sites for the pathogen and affecting fungal infection/colonisation of vascular tissue^2,3^, although the magnitude of this interaction may vary greatly with individual fungal strains^4^.

Chemical treatments, such as methyl bromide and chloropicrin, have been an indispensable tool for the past 40 years because of their excellent efficacy, effectively managing Verticillium wilt in strawberry; however, several of these treatments are already banned (e.g. methyl bromide) or face an uncertain future due to legislation (e.g. chloropicrin)^5^. With the loss of methyl bromide and other fumigants, strawberry (*Fragaria* × *annanasa*) production has come under increasing threat of losses due to wilt caused by *V. dahliae*. Some alternative measures have control efficacy similar to that achieved by commercial chemical fumigants, but others are not as good^6^. One of the alternatives being investigated is biofumigation, which uses decay products of green manures^7–10^. Control of *V. dahliae* through the use of *Brassica* species plants is believed to result from the toxic isothiocyanates (ITCs), released into the soil after incorporation of glucosinolate-containing plant tissues^11^. A recent study suggested that biofumigation based on the *Brassica* species cannot fully control wilt because of the limited amount of the ITC released^9^. Other plant species have also been studied for their biofumigation effects against *V. dahliae*. For example, biofumigation using *Lavandula* plant materials can result in large reductions in the numbers of viable microsclerotia recovered^12^. Recently, anaerobic soil disinfestation (ASD) methods have been studied for their effects against a range of soil pests and pathogens. Traditional ASD with grass was less effective than with organic materials; all materials proved to be effective at 16 °C against *P. penetrans*, *Meloidogyne hapla*, *Globodera pallida* and *V. dahliae* with *V. dahliae* being most difficult to control^13^. Control efficacy depends on many factors, including soil characteristics, types of organic material, temperature, dosage and exposure time. Soil organic amendments, especially combined with biocontrol agents, can satisfactorily suppress Verticillium wilt^14^, but the effect is often inconsistent and pathosystem specific, revealed by a meta-study^15^. Rotation with other crops, e.g. Brassica and lettuce, can reduce wilt on strawberry^16,17^, but this management strategy is generally not commercially viable.

Specific microbial organisms have been tested against soil-borne pathogens, including *V. dahliae*. Application of two biocontrol organisms (*Paenibacillus alvei* K165 and the nonpathogenic *Fusarium oxysporum* F2) at the transplant stage reduced Verticillium wilt symptom development in aubergine^18^. Both organisms induced the expression of the pathogenesis-related (PR) proteins PR1 and PR4 in the stem of aubergines. Many fungi and bacteria were isolated from rhizosphere of oilseed rape and strawberry and tested against *V. dahliae*^19,20^. Many bacterial (primarily *Pseudomonas* and *Serratia* spp.) and fungal species were found to be antagonistic against wilt. There was also some evidence to suggest that some fungi were specifically enriched in each rhizosphere, which is supported by a recent finding that rhizosphere communities are partially genetically controlled by hosts^21^. Dipping plants in a suspension of *Serratia plymuthica* prior to planting reduced Verticillium wilt of strawberry and increased yield^22^. Plant growth-promoting rhizobacteria (PGPR) have recently received much attention for their use to increase crop production, including their role in suppressing disease development^23^. Non-pathogenic strains of *V. dahliae* can also be used as a biological control agent to exploit the concept that preoccupation of the ecological niche rendered strawberry plants immune to infection with pathogenic *V. dahliae*. Inoculation of non-pathogenic strains of *V. dahliae* reduced wilt on 20% of treated plants but led to increased wilt development on 50% of treated plants^24^.

The monoterpenoids associated with the *Lavandula* spp., which are of lower volatility than the ITCs associated with brassica decomposition, were detected for more than one week after materials were incorporated in soil^12^. Three of these terpenoids were shown to reduce microsclerotium viability in microcosm tests. Recently, we have conducted field experiments to compare a new product based on microencapsulation of the three terpenes (cineole, camphor and borneol) and several other alternatives for their efficacy against wilt on strawberry with the commercial chemofumigant (chloropicrin) as a standard. Results from the trials showed that overall the terpene-based product reduced wilt development but did not increase fruit yield, compared to the un-treated control. However, at two of the eight trial sites, the chloropicrin treatment led to nearly 25% increase in yield, compared to all other treatments, but the level of wilt was similar among all treatments. Chloropicrin-treated plants had increased growth vigour compared to all other treatments; all other treatments had more or less uniformly stunted growth, which cannot be associated with any obvious biotic and abiotic factors (although strawberry roots were not sampled for assessment). We speculated that this yield decline resulted from interactions among a number of microbial organisms, similar to apple replant disease^25^. Involvement of other beneficial or pathogenic microbial organisms in strawberry was implicated in several other experiments on controlling Verticillium wilt^13,22^.

This paper reports the results from studies aiming to identify candidate microorganisms that are responsible for observed yield decline in strawberry of non-chloropicrin-treated plants. This study reports primarily data-driven research, aiming to generate hypotheses on the possible candidate organisms responsible for the observed yield decline, which can then be further tested in future. Specifically, we used an amplicon-based metagenomics approach to profile soils from different treatments at four sites – two with and two without yield-decline phenomenon. Through statistical comparison of individual microbial operational taxonomy units (OTUs), combined with several objective selection criteria, several candidate organisms were identified as candidates that may have played a role in the observed yield decline.

## Materials and methods

### Field sites and sample collection

Evaluation of the efficacy of a new microencapsulated terpene-based product and other alternatives to control Verticillium wilt was conducted at eight sites: three in 2011, three in 2012 and two in 2013. At two sites in 2012, a yield-decline phenomenon in strawberry was observed. Based on the physical distances between the trial sites, four sites (two in 2012: PV12 and HB12 and two in 2013: HB13 and EM13) were selected for microbial profiling to identify candidate microorganisms that may be involved in the yield-decline phenomenon. HB12 and HB13 were two fields at the same farm (about 1 km apart), about 15 km and 25 km from the PV12 and EM13 sites, respectively; PV12 was about 40 km from the EM13 site. At each site there were three blocks; within each block, there was one plot per treatment. The plot size was 15 m long (a single bed of double rows) with four metres between neighbouring plots. Immediately after soil treatment, all beds were covered with black polythene; beds were planted three weeks after treatment. All plots were automatically irrigated through drip tape.

Soil samples from the four sites were obtained in 2013 or 2014; details of samples are given in Table 1. Although there were six (2012) and eight (2013) treatments, only selected treatments were sampled for microbial profiling. For the two yield-decline sites, nearly two years had passed since the treatment when soil samples were taken in 2013. Thus, the chloropicrin effect in preventing yield decline may have significantly reduced – this had to be taken into account when trying to identify causal agents responsible for yield decline at these two sites. Although for the two 2012 sites, soil samples were taken long after the trial cropping was finished, the land was not used for other purposes and hence the plot (bed) structure was still intact at the time of sampling. For each plot, a composite soil sample was obtained, consisting of 10 core soils that were obtained with a sampler (2.5 cm in diameter) from a depth of 20 cm at randomly selected locations and then mixed by sieving (mesh size 2 mm). A subsample (approximately 2 g) of each composite sample was collected in a 2 ml Eppendorf tube and stored at −80 °C until DNA extraction.

### DNA extraction and next generation sequencing

Total genomic DNA was isolated in triplicate from each soil sample (0.25 g) using the PowerSoil DNA Isolation Kit (MoBio Laboratories) with minor modifications as described below. Before bead-beating, the samples were incubated in lysis solution at 65 °C for 10 min. Samples were homogenised by two 20-sec cycles at power setting of 5.0 in FastPrep instrument (FP120, Bio 101, Thermo Savant, Qbiogene), with 5 min on ice in between cycles. The DNA was further extracted according to the kit protocol. Triplicate samples were pooled after extraction and purified using GeneClean Turbo Kit (MP Biomedicals) using GNomic salt solution as protocol. After preliminary trial sequencing runs, two primer pairs were selected: one for bacteria (27F/534R 16S rDNA) and one for fungi (ITSI-F/Ek28-R 18S ITS). The two primer sets were modified at the 5′ end with adaptors, TCG TCG GCA GCG TCA GAT GTG TAT AAG AGA CAG – forward adaptor and GTC TCG TGG GCT CGG AGA TGT GTA TAA GAG ACA – reverse adaptor. The ITSI-F/Ek28-R primer set was chosen as it gave better amplification when combined with the barcode attachments used in the Illumina sequencing. PCR amplification using these primers gave a product of ∼750 bp, which was consistent with the target region of the rRNA genes including the end of the SSU, ITS1, 5.8S and the start of the LSU region plus the adaptor primers. All PCR reactions were carried out in triplicate 13.0 µl reactions with ×1 buffer basic (Molezym GmbH and Co. Bremen Germany), 2 mM MgCl_2_ (Qiagen, Hielden, Germany), 0.2 mM dNTP (Invitrogen, Life Technologies, USA), 0.25 U Mol Taq basic DNA polymerase (Molezym GmbH and Co. Bremen Germany), 0.2 µM forward and reverse primers each (Integrated DNA Technologies) and about 2 ng template DNA, was made up to 13 µl with molecular biology reagent water (Sigma, UK). Each reaction was performed in a Dyad thermocyler (MJ research), according to the following protocol, thermal cycling consisted of initial denaturation at 94 °C for 3 min, followed by 35 cycles of denaturation at 94 °C for 30 s, annealing at 55 °C for 45 s, and elongation at 72 °C for 60 s, reducing 0.5 °C per cycle until 50 °C, with a final extension at 72 °C for 5 min. Negative control samples were treated similarly with the exclusion of template DNA. PCR products were visualised by agarose gel electrophoresis.

Following PCR, DNA amplicons were purified using Agencourt AMPure XP beads (Beckman Coulter, USA), as per manufacturer’s instructions. The adapted amplicons were then modified by attaching indices and Illumina sequencing adapters using the Nextera XT Index Kit by PCR as described in the manufacturer’s protocol, enabling simultaneous sequencing of multiple samples, i.e. multiplexing. Following the index PCR clean-up step, using the Agencourt AMPure XP beads, as per manufacturer’s instructions, PCR products were qualitatively assessed using a Fragment Analyzer (Advanced Analytical, Ames, IA, USA) with the High Sensitivity NGS Fragment Analysis Kit (Advanced Analytical, Ames, IA, USA). PCR products were also quantitatively assessed using a Qubit 2.0 Fluorometer (Life Technologies, USA).

DNA from the different samples was then pooled so as to be analysed on the same Illumina run to avoid run-quality bias. The unique DNA barcode indices allowed sequences from all samples to be de-multiplexed in subsequent processing. Samples were pooled in such a way to ensure each of them was equimolar. The final concentration of the pooled library was 4 nM. The amplicon library was denatured using 1 mM NaOH and diluted to 30 pM as per manufacturer’s protocol. The diluted and denatured amplicon library was then combined with a denatured PhiX library at an equimolar concentration at a rate of 20% to increase heterogeneity of the sample. These samples were then run on an Illumina MiSeq with 300 bp paired end sequencing (version 3 chemistry). Samples from 2013 were sequenced separately from the 2014 samples; within each sequence run, there were 64 samples (which included samples from other studies).

### Sequence processing

Raw sequences were automatically de-multiplexed by the Illumina MiSeq and then further quality-filtered by the QIIME analysis pipeline:^26^ (1) removing primers from sequences, (2) removing low-quality reads, (3) identifying an OTU for each sequence against two international databases: 16S (bacteria) – Silva^27^ and the UNITE fungal 18S ITS database^28^ at 97% similarity, (4) storing every unique sequence and its frequency in each sample. Finally, we wrote a small utility programme in Delphi to (1) produce summary for OTU frequency for each sample, (2) merge the OTU frequency data over all samples and (3) produce an overall OTU table in the BIOM format, enabling further downstream statistical analysis.

We also customised the UNITE fungal ITS database to include ITS sequences for oomycetes and vascular wilts; all oomycete ITS sequences were first obtained from international repositories – if multiple sequences were available for a single species then a consensus sequence was generated with Geneious version 6.1 (Biomatters Ltd). Custom Perl scripts were written to query the Index Fungorum database (http://www.indexfungorum.org/), using the Index Fungorum Fungus API to query the website to return taxonomic information on the additional species, absent from the UNITE database. Both the consensus sequences and the linked taxonomic information were then appended to the UNITE database files. Full scripts are available for download from (https://github.com/eastmallingresearch/metagenomics).

### Statistical analysis

Two types of statistical analyses were performed: (1) initial exploratory analysis and (2) detecting differential OTU abundance between samples from different sites to identify candidates responsible for the yield-decline phenomenon.

Individual sample diversity (i.e., α diversity) was calculated: the number of distinct OTUs observed per sample (Sobs) and Shannon and Simpson indices (both related to the frequency of individual OTUs within a sample). To reduce biases in sequencing depth on these indices, a re-sampling (i.e. bootstrap) scheme was used to estimate α diversity indices for each sample. A bootstrap sample was obtained via randomly sampling a minimum number of sequences from the sequences in each sample (i.e., rarefying). All indices are calculated from each bootstrap sample at the rarefaction point – a total of 25 bootstraps were conducted. Mean indices were calculated from these bootstrap samples. Next, we calculated diversity indices among samples (i.e. β diversity): Morrison-Horn (MH), Bray-Curtis (BC) and ThetaYC (YC) indices. MH measures the similarity between two samples whereas the other two methods measure the dissimilarity between two samples. Both α and β diversity indices were calculated using the Win64 version of the Explicet software^29^. A principal component analysis was conducted to detect overall differences between samples using the STAMP programme^30^.

To assess differential OTU abundance among treatments, we used the DESeq2 statistical package in R^31^. DESeq2 was developed for comparing differential gene expressions but is equally applicable to analysis of metagenomic data. DESeq2 provides a new statistical fitting routine to account for variance heterogeneity often observed in sequence data; it uses the negative binomial distribution as an error distribution to compare abundance of each OTU between groups of samples in the framework of generalised linear modelling. This method was superior to other methods commonly used for this purpose^32^; the same study also showed that rarefying samples is inferior to not rarefying in identifying differences in OTU abundances but with correct distribution assumptions. Thus, in the present study, rarefying was not used when comparing OTU abundance. The median-of-ratios method^33^ was used to normalise the data to correct for unequal sequencing depth; this procedure was implemented as a default in DESeq2. To correct false discovery rate associated with multiple testing, DESeq2 uses the Benjamini-Hochberg (BH) adjustment^34^. In addition, DESeq2 also implemented an algorithm to adjust for large variability in log-fold changes for small counts. Candidate OTUs were selected at the 5% significance level (BH adjusted). Any OTU with the total number of reads across all the samples less than three was omitted from differential abundance testing.

In comparing OTU abundance, seven different comparisons were made, taking into account the potentially complex nature of candidate organisms, and the persistency of chloropicrin effect in relation to sampling time. The first five comparisons were between sites with and without the yield-decline phenomenon; only samples collected in 2014 were used for these five comparisons since they were sampled at the same time. In these comparisons, chloropicrin-treated soil samples from HB12 and PV12 were excluded because it is not certain whether the chloropicrin effect could last for more than two years. These five comparisons were:

- EM13/HB13 (12 samples) vs. HB12/PV12 (six samples);
- EM13 (six samples) vs. HB12 (three samples);
- EM13 (six samples) vs. PV12 (three samples);
- HB13 (six samples) vs. HB12 (three samples);
- HB13 (six samples) vs. PV12 (six samples).
- The four individual comparisons (b–e) are necessary since it is possible that yield decline may be caused by more than one organism; thus an overall comparison, as in (1), may not be able to reveal all possible candidates because of potential site-to-site variability in the abundance of candidate organisms. The final two comparisons were:
- 2013 chloropicrin (three samples) vs. other treatments (nine samples) at PV12;
- 2014 chloropicrin (three samples) vs. the untreated (three samples) at HB12.

For comparison (f), only 2013 samples were used because the sampling time elapsed was close to two years after the application of treatments at PV12, similar to the time elapsed for the 2014 samples at HB12.

## Results

### Sequencing quality

**Figure 1:**
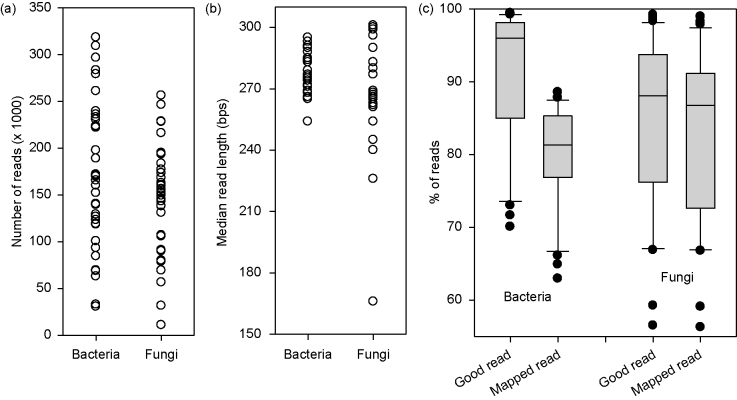
General information of sequencing data: (**a**) number of reads per sample, (**b**) median read length per sample and (**c**) % of good quality reads and % of reads mapped to OTUs.

Samples were sequenced in two runs: in February (2013 samples) and July (2014 samples), 2014. Most samples had more than 50 000 high-quality sequences (Figure 1). The median read length of the first read (P1), after quality trimming in the QIIME pipeline, was greater than 250 bps for all examples except four samples for the fungal sequencing (Figure 1). About 91% and 85% of total reads were of good quality for bacteria and fungi, respectively.

The majority of these high-quality reads are mapped to OTUs in the two international databases. For bacteria, on average 88% of reads were mapped to OTUs (ranging from 83% to 94% for individual samples). For fungi, the percentage of good quality reads mapped to OTUs ranged from 85% to 100% with mean of 96%. The total number of distinct bacterial and fungal OTUs was 2142 and 2022, respectively. Of these OTUs, there were respective 306 and 326 cases where there was only a single read across all samples, i.e. only present in one sample.

**Figure 2:**
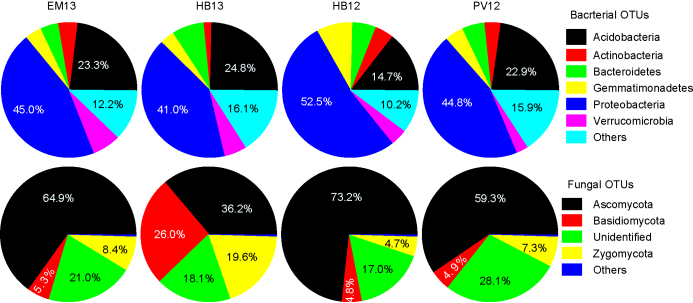
Proportion of major bacterial and fungal phylum found in the three untreated soil samples at each of the four sites; soils were sampled in May 2014.

The two most common bacterial phyla are Proteobacteria and Acidobacteria, accounting for ca. 67% of the total mapped reads (Figure 2). For fungi, Ascomycota was the most common phylum, accounting for more than 50% of the mapped reads on average, and the next two common phyla were Zygomycota and Basidiomycota (Figure 2).

**Figure 3:**
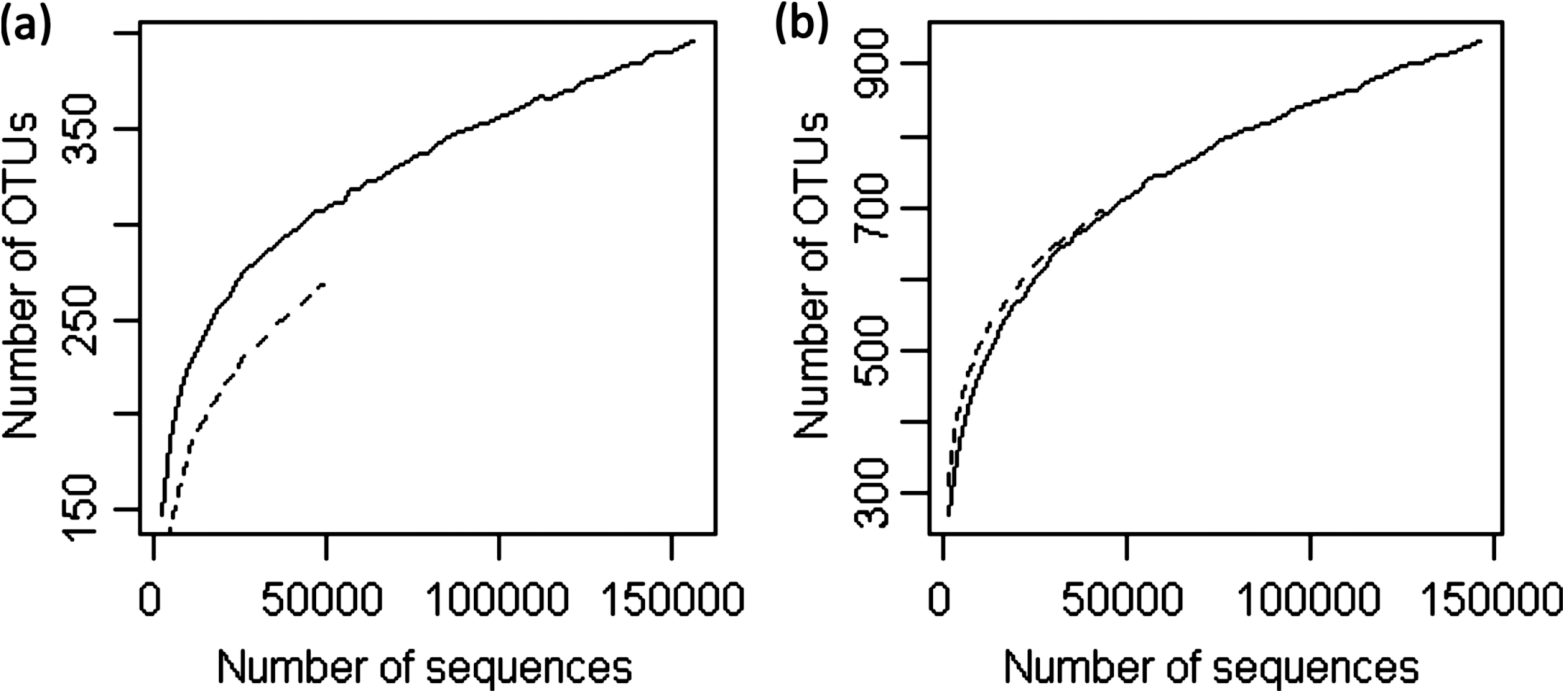
Four examples of rarefaction curves: two for fungi (**a**) and two for bacteria (**b**). All curves terminated at the number of sequences obtained in the examples.

The relationship between number of OTUs observed (Sobs) and the sequencing depth is shown in Figure 3 for a few samples. Overall, the present sequencing depth appeared to be sufficient since the minimum sampling depth in our samples was not in the part of curve with steep increases – indicating a diminishing return of further sequencing in uncovering new OTUs.

### α diversity

Within sample diversity measures varied greatly with samples (Table 2). Overall, there were more bacterial OTUs in individual samples than fungal OTUs (Table 3). At the rarefaction point (35 000 and 46 000 for bacteria and fungi, respectively), the number of bacterial OTUs ranged from 506 to 764 per sample, compared to the corresponding fungal value of 123–367. The α diversity of fungal OTUs varied more from sample to sample than bacterial OTUs. For instance, the Simpson index ranged from 0.054 to 0.50, compared to the corresponding value of 0.013–0.037 for bacteria. The effect of chloropicrin on the number of OTUs was inconsistent between sites. Only at the EMR site, chloropicrin appeared to consistently reduce the number of OTUs, compared to the control treatment (Table 2).

### β diversity

**Figure 4:**
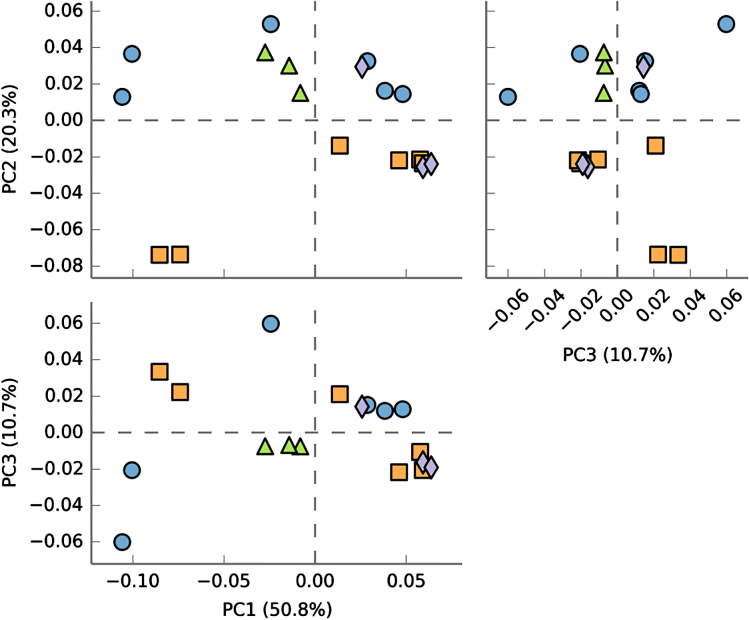
Pairwise plots of the first three principal components from a principal component analysis of the bacterial OTU data (together with % variance accounted by each component) for samples taken in May 2014 from four sites: EM13 (circle), HB12 (triangle), HB13 (square) and PV12 (diamond). For PV12 and HB12 sites where yield decline was observed for non-chloropicrin-treated plants, samples for the chloropicrin-treated plots were not included.

**Figure 5:**
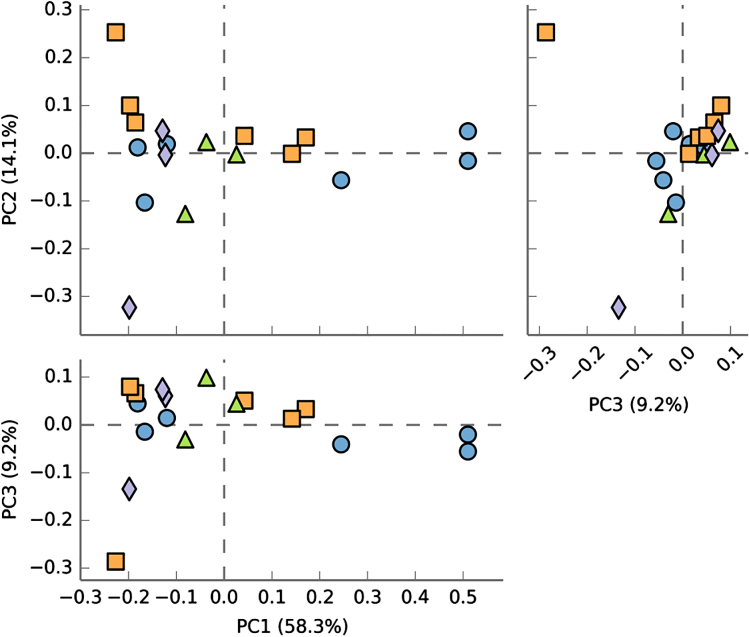
Pairwise plots of the first three principal components from a principal component analysis of the fungal OTU data (together with % variance accounted by each component) for samples taken in May 2014 from four sites: EM13 (circle), HB12 (triangle), HB13 (square) and PV12 (diamond). For PV12 and HB12 sites where yield decline was observed for non-chloropicrin-treated plants, samples for the chloropicrin-treated plots were not included.

Table 3 shows the estimated β diversity measures for bacteria and fungi. In general, β diversity among samples was greater for fungi (i.e. lower similarity, high dissimilarity) than for bacteria. However, these diversity estimates did not show consistent patterns regarding their relationship with site and yield decline. For example, EMR site (no yield decline) showed the least similarity to the two sites with the yield-decline phenomenon (HB12 and PV12). However, HB12 was the least similar to PV12. Samples from HB12 and PV12, as a group, were not clearly separated from other samples based on principal component analysis of all bacterial OTUs (Figure 4) or fungal OTUs (Figure 5).

### Differential abundance

**Figure 6:**
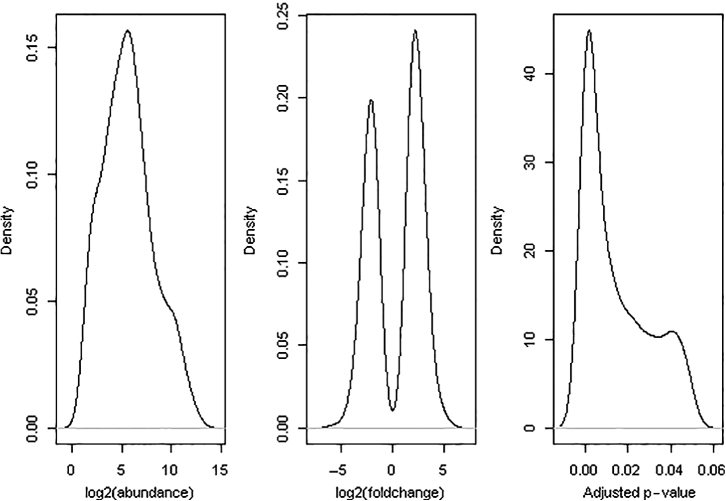
Density plots of three statistics for 591 cases for which there were significant differences in bacterial abundance between non-yield-decline and yield-decline samples: overall OTU abundance across all samples, log2-fold changes and BH-adjusted *p*-values.

**Figure 7:**
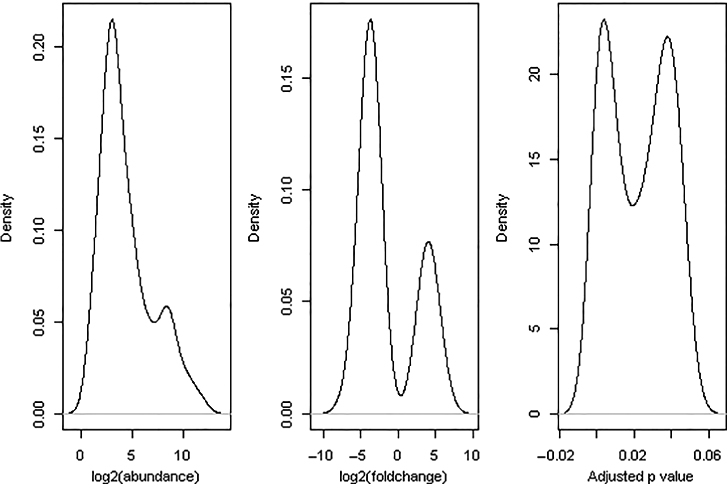
Density plots of three statistics for 124 cases for which there were significant differences in fungal abundance between non-yield-decline and yield-decline samples: overall OTU abundance across all samples, log2-fold changes and BH-adjusted *p*-values.

A total of 452 bacterial and 485 fungal OTUs were omitted from differential abundance testing because of extreme low counts across all samples (total counts ≤ 3), leaving 1690 bacterial and 1537 fungal OTUs for statistical testing. Of all pairwise comparisons, 715 comparisons were statistically significant at the 5% level. Figures 6 and 7 show the density plots of three statistics for these significant comparisons: average abundance for the OTUs, log2-fold change (no-yield-decline samples over yield-decline samples) and BH-adjusted *p*-values. Overall, more bacterial OTUs appeared to be more abundant in the non-yield-decline soils than in the yield-decline soils; the opposite was true for fungal OTUs.

There were 591 and 124 significant comparisons for bacterial and fungal OTUs, respectively. Excluding multiple significant comparisons for a single OTU, there were 366 and 88 unique bacterial and fungal OTUs for which there were significant differences in their abundance between the two types of samples (yield-decline vs. non-yield-decline). These fungal OTUs did not include common soil-borne pathogens, e.g. *Fusarium*, *Verticillium*, *Phytophthora* and *Pythium*. The log2-fold change was greater and less than zero for 326 and 325 bacterial comparisons, respectively; the corresponding values for fungal OTUs were 38 and 86. For bacteria, there were 203, 112, 35, 11 and 3 OTUs for which one, two, three, four and five out of the seven comparisons were statistically significant, respectively. For fungi, there were 63, 15, 9 and 1 OTUs for which one, two, three and four out of the seven comparisons were statistically significant, respectively.

To narrow down the number of OTUs for further interpretation of their possible roles in affecting yield decline, the following criteria were applied to these 454 OTUs (366 bacteria and 88 fungi):

1. average abundance should be over 10 and 6 across all samples for bacterial and fungal OTUs, respectively; this was used to exclude those OTUs with low counts since it is reasonable to assume that a high level of an OTU is needed if it was partially responsible for causing yield-decline;
2. the absolute log2-fold change is greater than 2.0 (i.e. the difference in abundance is at least four-fold);
3. there should be at least two statistically significant comparisons (out of the seven) for one single OTU;
4. for a single OTU, its effect sign (i.e. negative or positive) must be consistent among those significant comparisons involving the OTU.

In total, 32 bacterial and 17 fungal OTUs met these criteria. Of the 32 bacterial OTUs, log2-fold change was positive for 24 cases, i.e. no-yield-decline samples had greater abundance than the yield-decline samples. In 13 of the 17 fungal OTUs, no-yield-decline samples had lower abundance than yield-decline samples. Statistical test showed that there was significant differential abundance between fungal and bacterial OTUs between the two types of samples (*p* < 0.001).

Table 4 gives the taxonomical information of these 32 bacterial and 17 fungal OTUs. Of the 32 bacterial OTUs, 16 were from the phylum of Proteobacteria and six from Firmicutes. The remaining 10 OTUs were from eight phylum groups (Table 4). Ten fungal OTUs were from the Ascomycota and five from Basidiomycota. Further analysis of these OTUs in Table 4 (based on published research studies or online information) suggested 12 bacterial and 4 fungal OTUs (Table 5) could have played a role in the yield-decline phenomenon. Yield decline may result from one or more (and/or their interactions) of the following four factors: (1) lack of beneficial bacteria, (2) lack of nematode-parasitic fungi, (3) high levels of non-specific fungal root rot pathogens and (4) wet soil conditions.

**Figure 8:**
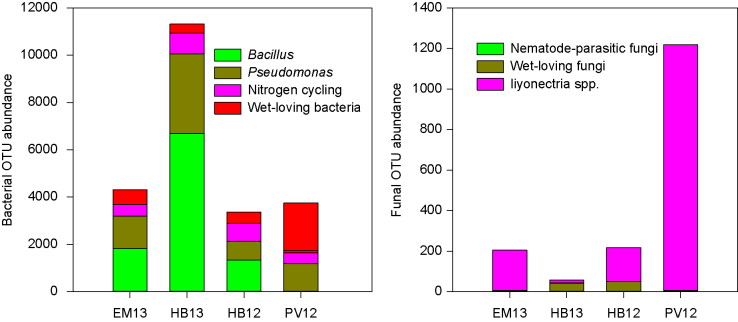
Relative abundance of the 16 (12 bacterial and 4 fungal OTUs – Table 7) organisms per sample that may have played a role in affecting strawberry yield decline observed at HB12 and PV12; counts data were proportionally adjusted to the total reads of 300 000 per sample. Samples from chloropicrin-treated plots at HB12 and PV12 were excluded.

Figure 8 shows the relative abundance of these 16 candidate OTUs at the four sites. HB13 and EM13 had much higher levels of *Bacillus* and *Pseudomonas* OTUs than HB12 and PV12. The level of wet-loving bacterial OTUs was higher at PV12 than at the other sites; the opposite was true for the level of bacterial OTUs related to nitrogen cycling. For fungal OTUs, the level of two *Ilyonectria* species was the highest and lowest at PV12 and HB13, respectively. There was a higher level of wet-loving fungi at HB12 than at the other three sites. Nematode-parasitic fungi were nearly exclusively found at HB13.

## Discussion

The amplicon-based metagenomic analysis of soil samples utilised in this study identified several groups of microbial organisms that may be involved in causing strawberry yield decline. Many OTUs differ in their abundance between samples from the yield decline and non-decline soils. Based on the comparisons of abundance of each individual OTU and several stringent criteria, we identified up to 51 (34 bacterial and 17 fungal OTUs) that were most likely to be involved in affecting yield decline of strawberry. Of these 51 OTUs, only for 12 bacterial OTUs and four fungal OTUs is there published information about their possible roles (or those of closely related species), which is biologically plausible to explain why the OTUs were implicated in strawberry yield decline.

There are two fungal OTUs that had very high abundance at one of the two yield-decline sites (PV12); these two OTUs are *Ilyonectria robusta* and *I. coprosmae*. Of these two OTUs, *I. robusta* is particularly abundant at PV12. Based on recent molecular taxonomy, these two *IIyonectria* species are closely related to *Cylindrocarpon* spp; indeed, *IIyonectria* contains many *Cylindrocarpon*-like species that have been commonly associated with root and decay of woody and herbaceous plants^35^. Recently, *I. robusta* has been shown to cause root diseases on grapevine (including vascular invasion)^36–38^. *Cylindrocarpon*-like and *Ilyonectria* cause diseases on Laurustinus^39^. Potential fungal pathogens other than *V. dahliae* were frequently recovered from strawberry roots, e.g. *Rhizoctonia* sp.^40,41^, and *Cylindrocarpon destructans*, *Fusarium oxysporum*, *Fusarium solani*, *Pestalotia longiseta* and *Pythium* spp^42–44^. *Cylindrocarpon destructans* can cause variable degrees of crown and root rot in strawberry^45,46^. Collectively these general, non-specific pathogens that cause a root disease are commonly referred to as black root rot, a name that is descriptive of the appearance of the roots^47^.

The importance of these non-specific root pathogens may have been masked by two factors. First, broad-spectrum chemo-fumigants (e.g., methyl bromide and chloropicrin) have been used to fumigate soil. Second, unlike *V. dahliae* these non-specific root pathogens do not usually lead to plant mortality but only lead to reduced plant growth. This reduced plant growth is often difficult to differentiate from nutrient deficiency or other abiotic factors. In non-fumigated soils, strawberry yield reduction between 20–25%^43^ and 46% (http://www.mbao.org/altmet00/32martin.pdf, accessed on 26 November 2014) was observed but was not attributable to *V. dahliae*, similar to the loss observed at HB12 and PV12 (ca. 25%). Based on root isolations, this yield decline was attributed to root rot caused by *Pythium*, binucleate *Rhizoctonia* and *Cylindrocarpon* spp.

Of the 12 bacterial OTUs, 2 were from the genus *Bacillus* and 2 from the genus *Pseudomonas*, which were more abundant in non-yield-decline soil samples. Many strains from *Bacillus* and *Pseudomonas* have been demonstrated to have anti-fungal effects and can promote plant growth^48–56^. There are already several commercial products based on the strains from these two genera marketed as biocontrol products against plant diseases and/or as PGPR products. Probably the most widely used biocontrol strain for plant pathogens is *B. subtlis*, e.g. one formulated commercial product is called Serenade®. Soil suppressiveness against non-specific root pathogens is associated with the high level of total fungi and fluorescent bacteria^42^; suppressiveness is reduced on sites where a strawberry monoculture without organic input has been grown for several years. Two *Bacillus* strains reduced ginseng root rot caused by *C. destructans*^57^. Several *Pseudomonas* strains isolated from the rhizosphere of oilseed rape and strawberry were antagonistic against *V. dahliae*^19^. Early and localised root surface and root endophytic colonisation by *P. fluorescens* PICF7 is needed to impair full progress of Verticillium wilt epidemics in olive^58^. However, these identified beneficial bacterial OTUs in the present study are not likely to have much antagonistic effect against *V. dahliae* since the wilt incidence was similar between yield-decline and non-yield-decline plots at HB12 and PV12.

At the HB13 site, an OTU from the genus of *Paecilomyces* was much greater in abundance than at the two yield-decline sites. This genus is known to contain nematophagous fungal species, killing nematodes by pathogenesis^59–61^. It is also generally accepted that nematodes may exacerbate wilt problems in strawberry by providing wounds as fungal entry sites. The presence of nematodes increased the rate of Verticillium wilt development on strawberry except when the fungal inoculum level was very low^62^. In the presence of *V. dahliae* inoculum, the tolerance of strawberry to lesion nematodes was reduced by 50%^63^. Wilt in potatoes is more severe in the presence of lesion nematodes^64^.

Five bacterial OTUs were more abundant in non-yield-decline soils than in yield-decline soils. They are reported to have been involved in global nitrogen cycles, e.g., Methylophilaceae – de-nitrification^65^, Rhizobiaceae – nitrogen fixation^66^ and Nitrosomonadaceae – nitrification (oxidising ammonia into nitrite)^67^. Thus, these organisms may improve plant growth due to increased availability of nitrogen in the soil to plants. Several other microbial OTUs were more abundant in yield-decline soils than in non-decline soils and they are usually abundant in water or wet conditions or involved in anaerobic respiration. These OTUs are not likely to directly influence strawberry growth. Rather their abundance may indicate water logging or high levels of soil moisture content, which in general would favour pathogen development and reduce root development in strawberry^68^. Soil physiochemical properties may also affect plant and pathogen development. It is not always clear whether such effects are through their direct influence on plant development or (partially) mediated through their effects on soil microbial populations.

The two yield-decline sites have been in continuous strawberry production for many years. In contrast, HB13 site was previously an apple orchard and EM13 site was planted with cereals in previous years. Rotation is one of the cultural practices used to manage diseases, particularly soil-borne pathogens^69–72^. Rotation was able to reduce the severity of strawberry root rot^73–75^. Continuous cropping of a single species for a long period of time often leads to reduced cropping potential in rosaceous species, e.g., apple^25^ and almond^76^. Further research is needed to understand which bacterial and fungal OTUs associated with strawberry yield decline are also associated with continuous cropping of strawberry and which of these OTUs are due to the nature of soils.

Soil sampling in the present work was unfortunately constrained by the limited number of sites with yield-decline and commercial horticulture. Thus it was not possible to adopt a more controlled sampling plan that could adequately consider other site-specific factors, such as soil type, previous crops and host genotypes. These site-specific factors undoubtedly affect plant health, either contributing to soil disease suppression and nutrient supply/uptake or predisposing plants to pathogens^77,78^. However, they are unlikely to be the determinant of strawberry yield decline because strawberry plants in plots treated with chloropicrin at the same sites did not suffer from the yield decline. The present study, based on the data-driven approach, was able to identify candidate microbes for further studies where some of these site-specific factors could be included. For instance, how do the two candidate fungal pathogens interact with beneficial soil microbiota in different types of soil in relation to strawberry yield?

It should be noted that the present approach (relative DNA abundance) will not be applicable to scenarios where a very low level of microbes could cause disproportional effects on plant development, e.g., by producing toxins. Such a problem (linking soil microbial functions to plant development) may potentially be dealt with metatranscriptomics (RNA-seq) or metabolomics-based approaches. Recent research reports on the transcriptomes of microbial community in soil^79^ and Arctic peat soil^80^ highlight the feasibility of applying metatranscriptomics to soil microbes (though still challenging).

In summary, this study demonstrated the usefulness of amplicon-based metagenomics to identify candidate organisms involved in affecting strawberry yield decline. Isolation and culture techniques are usually used to obtain microbial organisms from root systems for further pathological studies. But this time-consuming approach suffers from the fact that most microbial organisms cannot be cultured in artificial media. An amplicon-profiling approach provided an efficient way to profile microbiota and identify candidate organisms for further hypothesis testing and confirmatory studies.

## Figures and Tables

**Table 1:** Summary of soils sampled for metagenomic profiling; there were three composite samples (one from each replicate plot) for each treatment at each site, giving a total of 36 samples

				Treatments sampled		Last harvest time
Site	Yield decline?	Cultivar	Treatment applied	October 2013	May 2014	
EM13	No	Elsanta	May 2013	Not sampled	Chloropicrin, untreated	14 July
HB13						
HB12	Yes	Elsanta	May 2012	Not sampled	Chloropicrin, untreated	13 July
PV12		Eves Delight	November 2011	BF^a^, chloropicrin, LW^a^, untreated		12 October

a Coded products – the exact identity is commercially confidential.

**Table 2:** Summary of α (within-group) diversity measures calculated at the rarefaction point (35 000 and 46 000 for the bacteria and fungi, respectively): number of OTUs (Sobs), Shannon H index (SH), Shannon E index (SE), Simpson Index (SI)

			Bacteria				Fungi			
Year	Site	Treatment	Sobs	SH	SE	SI	Sobs	SH	SE	SI
2013	PV12	BF	587	6.570	0.714	0.033	266	3.620	0.449	0.228
2013	PV12	BF	602	6.780	0.734	0.026	236	4.012	0.509	0.157
2013	PV12	BF	599	6.697	0.725	0.024	231	3.464	0.442	0.207
2013	PV12	Untreated	586	6.562	0.714	0.033	287	4.481	0.549	0.105
2013	PV12	Untreated	551	6.273	0.689	0.037	269	4.653	0.576	0.088
2013	PV12	Untreated	620	6.756	0.729	0.026	249	3.373	0.424	0.278
2013	PV12	Chloropicrin	600	6.715	0.728	0.025	180	2.705	0.361	0.342
2013	PV12	Chloropicrin	583	6.690	0.728	0.024	203	3.724	0.485	0.168
2013	PV12	Chloropicrin*					237	3.840	0.487	0.202
2013	PV12	LW	635	6.898	0.740	0.023	205	4.581	0.596	0.080
2013	PV12	LW	657	7.093	0.758	0.020	226	4.200	0.537	0.115
2013	PV12	LW*								
2014	EM13	Untreated	629	6.679	0.718	0.024	284	4.924	0.604	0.062
2014	EM13	Untreated	654	6.800	0.727	0.023	349	4.959	0.587	0.070
2014	EM13	Untreated	641	6.704	0.719	0.026	246	5.079	0.640	0.054
2014	EM13	Chloropicrin	506	6.362	0.709	0.028	169	2.236	0.303	0.500
2014	EM13	Chloropicrin	545	6.358	0.699	0.025	154	2.026	0.279	0.491
2014	EM13	Chloropicrin	572	6.569	0.717	0.020	181	3.028	0.403	0.229
2014	HB12	Untreated	664	7.006	0.747	0.016	326	4.951	0.593	0.073
2014	HB12	Untreated	672	6.913	0.736	0.018	323	4.559	0.547	0.100
2014	HB12	Untreated	632	6.831	0.734	0.019	350	4.956	0.587	0.078
2014	HB12	Chloropicrin	639	6.900	0.741	0.017	208	3.893	0.505	0.186
2014	HB12	Chloropicrin	649	7.044	0.754	0.014	246	4.243	0.535	0.128
2014	HB12	Chloropicrin	576	7.101	0.775	0.013	304	5.026	0.610	0.076
2014	HB13	Untreated	666	6.774	0.722	0.024	367	5.064	0.594	0.075
2014	HB13	Untreated	576	6.804	0.743	0.022	289	4.763	0.583	0.093
2014	HB13	Untreated*	656	6.761	0.723	0.025				
2014	HB13	Chloropicrin	613	6.634	0.716	0.024	159	3.595	0.492	0.173
2014	HB13	Chloropicrin	639	6.711	0.720	0.023	284	4.098	0.503	0.150
2014	HB13	Chloropicrin	645	6.705	0.718	0.024	204	4.340	0.566	0.100
2014	PV12	Untreated	664	6.981	0.745	0.024	261	3.396	0.423	0.208
2014	PV12	Untreated	651	6.608	0.706	0.026	347	5.238	0.621	0.060
2014	PV12	Untreated	654	6.767	0.724	0.027	202	5.024	0.656	0.054
2014	PV12	Chloropicrin	713	7.091	0.748	0.016	303	4.794	0.582	0.081
2014	PV12	Chloropicrin	735	6.861	0.721	0.025	349	5.032	0.596	0.072
2014	PV12	Chloropicrin	764	7.197	0.751	0.017	339	5.309	0.631	0.059

**Table 3:** Estimates of three β diversity (between-group) measures for bacterial and fungal OTUs, describing the extent of similarity or dissimilarity among combinations of years and sites

	Bacteria					Fungi				
	EM13	HB13	HB12	2013 PV12	2014 PV12	EM13	HB13	HB12	2013 PV12	2014 PV12
Morrista-Horn (similarity)										
EM13	1	0.856	0.934	0.77	0.826	1	0.74	0.756	0.361	0.486
HB13		1	0.834	0.886	0.89		1	0.806	0.71	0.633
HB12			1	0.745	0.802			1	0.573	0.738
2013 PV12				1	0.97				1	0.695
2014 PV12					1					1
ThetaYC (dissimilarity)										
EM13	0.000	0.251	0.123	0.374	0.297	0.000	0.413	0.392	0.78	0.679
HB13		0	0.285	0.205	0.198		0	0.325	0.45	0.537
HB12			0	0.407	0.33			0	0.598	0.415
2013 PV12				0	0.058				0	0.467
2014 PV12					0					0
Bray-Curtis (dissimilarity)										
EM13	0	0.279	0.316	0.412	0.586	0	0.484	0.462	0.649	0.505
HB13		0	0.358	0.302	0.564		0	0.439	0.492	0.609
HB12			0	0.394	0.442			0	0.632	0.493
2013 PV12					0.372				0	0.582
2014 PV12					0					0

**Table 4:** Bacterial and fungal OTUs that show significant differential abundance in soil samples from two types of soils: strawberry yield decline was or was not observed

Phylum	Class	Order	Family	Genus	Species	Effect sign
*Bacteria*						
Acidobacteria	Solibacteres	Solibacterales	(Bryobacteraceae)	Unidentified	Unidentified	+
″	Actinobacteria	Actinomycetales	Propionibacteriaceae	Unidentified	Unidentified	−
Bacteroidetes	(Saprospirae)	(Saprospirales)	Chitinophagaceae	*Chitinophaga*	Unidentified	+
″	Cytophagia	Cytophagales	Cytophagaceae	*Hymenobacter*	Unidentified	−
Chlorobi	Unidentified	Unidentified	Unidentified	Unidentified	Unidentified	+
Chloroflexi	Anaerolineae	Anaerolineales	Anaerolinaceae	*Anaerolinea*	Unidentified	−
Elusimicrobia	Elusimicrobia	FAC88	Unidentified	Unidentified	Unidentified	+
Fibrobacteres	Fibrobacteria	258ds10	Unidentified	Unidentified	Unidentified	+
Firmicutes	Bacilli	Bacillales	(Exiguobacteraceae)	Unidentified	Unidentified	+
″	″	″	Bacillaceae	*Bacillus*	*flexus*	+
″	″	″	″	″	Unidentified	+
″	″	″	Planococcaceae	*Solibacillus*	Unidentified	+
″	″	″	″	Unidentified	Unidentified	+
″	Clostridia	Clostridiales	Ruminococcaceae	Unidentified	Unidentified	−
Gemmatimonadetes	Gemmatimonadetes	Gemmatimonadales	Ellin5301	Unidentified	Unidentified	+
OD1	ABY1	Unidentified	Unidentified	Unidentified	Unidentified	−
Proteobacteria	Alphaproteobacteria	Caulobacterales	Caulobacteraceae	*Asticcacaulis*	*biprosthecium*	+
″	″	Caulobacterales	Caulobacteraceae	Unidentified	Unidentified	+
″	″	Rhizobiales	Bradyrhizobiaceae	*Bosea*	*genosp.*	+
″	″	Rhizobiales	Rhizobiaceae	*Agrobacterium*	*sullae*	+
″	″	Rhodospirillales	Rhodospirillaceae	*Skermanella*	Unidentified	−
″	″	Rickettsiales	mitochondria	Unidentified	Unidentified	+
″	″	Sphingomonadales	Sphingomonadaceae	*Novosphingobium*	Unidentified	+
″	Betaproteobacteria	Methylophilales	Methylophilaceae	*Methylotenera*	*mobilis*	+
″	″	Methylophilales	Methylophilaceae	Unidentified	Unidentified	+
″	″	Nitrosomonadales	Nitrosomonadaceae	Unidentified	Unidentified	+
″	Deltaproteobacteria	Desulfuromonadales	Geobacteraceae	*Geobacter*	Unidentified	−
″	″	Desulfuromonadales	Pelobacteraceae	Unidentified	Unidentified	−
″	Gammaproteobacteria	Alteromonadales	211ds20	Unidentified	Unidentified	+
″	″	Pseudomonadales	Moraxellaceae	Unidentified	Unidentified	+
″	″	″	Pseudomonadaceae	*Pseudomonas*	Unidentified	+
″	″	″	″	Unidentified	Unidentified	+
*Fungi*						
Ascomycota	Dothideomycetes	Capnodiales	Davidiellaceae	*Cladosporium*	C._sp_234B	−
″	″	″	Teratosphaeriaceae	*Devriesia*	*fraseriae*	+
″	″	Incertae_sedis	Pseudeurotiaceae	*Pseudeurotium*	*bakeri*	−
″	Eurotiomycetes	Eurotiales	Trichocomaceae	*Paecilomyces*	GZU_BCECYN4_3	+
″	Leotiomycetes	Helotiales	Incertae_sedis	*Tetracladium*	T._sp_WMM_2012d	−
″	Sordariomycetes	Hypocreales	Incertae_sedis	*Ilyonectria*	*coprosmae*	−
″	″	″	″	″	*robusta*	−
″	″	Microascales	Microascaceae	*Scedosporium*	*minutispora*	+
″	″	Microascales	Unidentified	Unidentified	M._sp_MCJA12	−
″	″	Unidentified	Unidentified	Unidentified	Sordariomycetes_sp	−
Basidiomycota	Agaricomycetes	Agaricales	Unidentified	Unidentified	uncultured_Agaricales	−
″	Microbotryomycetes	Leucosporidiales	Leucosporidiaceae	*Leucosporidiella*	fragaria	−
″	Tremellomycetes	Tremellales	Incertae_sedis	*Cryptococcus*	carnescens	+
″	″	″	″	″	macerans	−
″	Unidentified	Unidentified	Unidentified	Unidentified	Unidentified	−
Unidentified	Unidentified	Unidentified	Unidentified	Unidentified	fungal_sp_229c4c	−
Zygomycota	Incertae_sedis	Mortierellales	Mortierellaceae	*Mortierella*	Mortierella_sp_1_61D	−

**Table 5:** Summary of differential abundance between non-yield-decline and yield-decline samples and had met several other selection criteria (see the text) for a number of bacterial and fungal OTUs, together with the log2-fold change (LFC) and possible roles they played in the yield decline of strawberry, which was inferred from published studies and/or from online resources

			Significant comparisons		
Family	Genus	Species	ID^a^	Mean LFC	Possible roles
*Bacteria*					
Bacillaceae	*Bacillus*	*flexus*	abc	2.66	Biocontrol, PGPR^b^
		Unidentified	ade	2.80	Biocontrol, PGPR
Bradyrhizobiaceae	*Bosea*	*genosp*.	cfg	2.17	Nitrogen fixation
Rhizobiaceae	*Agrobacterium*	*Sullae*	abcde	3.38	Nitrogen fixation
Rhodospirillaceae	*Skermanella*	Unidentified	deg	−2.79	Usually in anaerobic aquatic environments
Methylophilaceae	*Methylotenera*	*mobilis*	aceg	3.26	Nitrogen cycling in natural environments
Methylophilaceae	Unidentified	Unidentified	acef	3.78	Nitrogen cycling in natural environments
Nitrosomonadaceae	Unidentified	Unidentified	cfg	2.22	Oxidize ammonia into nitrite
Geobacteraceae	*Geobacter*	Unidentified	cfg	−2.21	Anaerobic respiration
Pelobacteraceae	Unidentified	Unidentified	cef	−2.52	Anaerobic respiration
Pseudomonadaceae	*Pseudomonas*	Unidentified	acdeg	2.91	PGPR
	Unidentified	Unidentified	ace	3.14	PGPR
*Fungi* (*Ascomycota*)					
Trichocomaceae	*Paecilomyces*	GZU_BCECYN4_3	de	3.66	Killing nematodes
Incertae_sedis	*Tetracladium*	WMM_2012d	bd	−2.91	Water-logging rich fungus
Incertae_sedis	*Ilyonectria*	*coprosmae*	ace	−4.99	Including *Cylindrocarpon* spp. or *Cylindrocarpon*-like fungi that can cause non-specific root rot
		*robusta*	ae	−5.18	

a Identity of individual comparisons for which differences in microbial abundance were statistically different; a – EM13/HB13 vs. HB12/PV12; b – EM13 vs. HB12; c – EM13 vs. PV12; d – HB13 vs. HB12; e – HB13 vs. PV12; f – chloropicrin-treated vs. others at PV12 (2013 samples); and g – chloropicrin-treated vs. control at HB12.

b PGPR, plant growth-promoting rhizobacteria.
